# Herd-, sow-, parity- and service-level risk factors for sow mortality at different reproductive stages: an analysis of production data

**DOI:** 10.1186/s12917-026-05798-w

**Published:** 2026-08-15

**Authors:** Junhao Huang, Osvaldo Fonseca, Lola Pailler, M. Lorena Correa-Abendaño, Antonio Martínez, Jose Murillo, Carles Vilalta

**Affiliations:** 1 Centre for Research in Animal Health (CReSA), IRTA-UAB Joint Research Unit in Animal Health, Campus of the Autonomous University of Barcelona (UAB), Bellaterra, Catalonia Spain; 2 IRTA, Animal Health Program, Centre for Research in Animal Health (CReSA), Campus of the Autonomous University of Barcelona (UAB), Bellaterra, Catalonia Spain; 3 Vall Companys, Lleida, Spain

**Keywords:** Parity, Reproductive performance, Farrowing risk, Stillbirths, Mummified fetuses, Gilt management, Herd longevity, Sow death causes

## Abstract

**Background:**

Sow mortality has been increasing in recent years, posing significant challenges to commercial pig production. High mortality affects animal welfare, farm productivity, and causes substantial economic losses. While previous studies have identified risk factors such as high parity, seasonal effects, and farrowing-related complications, limited research has quantified mortality risk at different reproductive stages in Spain. This study aims to analyze sow mortality patterns and identify associated risk factors.

**Results:**

Analysis of production records from 16 Spanish farms revealed that 55.59% of sow deaths occurred before farrowing, while 44.41% occurred after farrowing. Mortality risk peaked during late gestation (105–118 days) and again during the first and after the fourth weeks post-farrowing.

Several risk factors were identified at farm and individual levels. Internal gilt replacement was associated with increased mortality risk. Before farrowing, sows with parity ≥ 3 and single-service sows exhibited higher mortality. After farrowing, increased mortality was observed in parity 1 sows and those with repeat breeding. Seasonal effects showed highest mortality rates in sows bred in spring and farrowing in summer.

Matched case-control analysis revealed that parity 0 sows had a younger age at first service, and deceased sows had shorter gestational length, fewer piglets born, born alive, and a higher incidence of stillbirth fetuses at their last litter before their death. Comparing their lifetime performance, dead sows had younger gilt age at first service, fewer parity at removal, higher average number of piglets born, born alive, born still and born mummified per parity, but they had fewer weaned piglets and nonproductive days.

**Conclusions:**

This study identifies late gestation and the first week postpartum as critical windows for sow mortality risk. Internal gilt replacement and parity extremes emerged as key herd- and sow-level risk factors, while mortality was also higher among sows farrowing in summer. Repeat breeding prior to farrowing was associated with lower mortality. These findings support integrating reproductive history and herd-level replacement strategy into sow management and culling decisions.

## Background

The increasing mortality rate of breeding sows has been rising at an alarming rate in recent years, with significant implications for various aspects of farm operations [1, 2]. In herds across the USA, Canada, Australia, and the Philippines, sow mortality reached 13.56% in 2021, following a steady rise from 7.32% to 11.78% between 2012 and 2018 [3]. In Spain, a study of over 100 farms reported that sow mortality rose steadily from approximately 8% in 2015 to 14–15% by 2024 [4]. This upward trend in sow mortality has resulted in productivity losses, particularly as a large proportion of these deaths occur during gestation, leading to significant piglet losses per sow per year.

The financial burden associated with sow mortality extends beyond direct losses, encompassing replacement costs, reduced reproductive output, and potential disruptions in herd management. Replacing a deceased sow can be costly, especially when gilt availability is limited, sometimes necessitating the retention of sows that would otherwise be culled. Additionally, gestational losses further amplify economic setbacks, with total expenses per lost sow estimated at $400–$500 [5]. A sow reaches positive net income around the third parity [6], and profit per litter rises with each subsequent parity by $100–$200 [7]. Thus, early loss or culling leads to considerable economic loss, with studies estimating an accumulated profit gap of $360–$441 between sows producing three and six litters [7]. Beyond its direct economic impact, sow mortality has also become a focal point of growing public and consumer concern regarding animal welfare in intensive livestock systems [8], making its reduction important not only for production efficiency but also for the broader social acceptance of animal agriculture.

Herd-level risk factors for sow mortality are influenced by a range of biological and environmental elements. Key factors include farm-specific conditions like housing, nutrition, and health management [9–13]. Feeding practices influence sow mortality by affecting digestive and metabolic stress [14]. High ambient temperature and humidity markedly increase sow mortality by overwhelming heat-dissipation mechanisms, leading to cardiovascular failure, especially during poorly ventilated, humid summer periods without nighttime cooling or acclimatization [15]. Biosecurity management is a key determinant of infectious disease occurrence. Among swine diseases, porcine reproductive and respiratory syndrome virus (PRRSV) is one of the most critical, raising the death rate by approximately 14% compared with naïve or stable breeding herds [16], causing an estimated annual economic loss of $380.82 million in the U.S. breeding sector from 2016 to 2020 [17].

Sow-level factors include sow characteristics such as reproductive performance, age at first farrowing, and parity, along with genetic breed [2, 3]. Sows with a younger age at first farrowing and larger first-litter size were found to have longer lifespans [18]. In high-parity sows, mortality is elevated from torsions of abdominal organs, prolapse, and cystitis-pyelonephritis, while gilts and first-parity sows are vulnerable due to physiological immaturity, pelvic constraints, and stress associated with first estrus, mating, and parturition [19]. Breed affects sow mortality through susceptibility to PRRSV infection [20], viral replication in alveolar macrophages [21], gut microbiota composition [22], and immune response capacity [23, 24].

Sows undergo marked physiological and metabolic changes across reproductive stages, particularly between gestation and the post-farrowing period [25, 26]. During late gestation and early lactation, sows experience substantial shifts in energy balance, endocrine regulation, immune function, and physical load [26]. Previous studies have reported different distributions of diseases across reproductive stages. For example, lameness was reported of showing differing prevalence or incidence patterns across gestation and lactation [27–29]. Similar stage-dependent patterns have also been observed for other disorders, including reproductive complications, metabolic diseases, and infectious conditions [30, 31]. As established contributors to sow mortality, these conditions may contribute to the previously reported variation in the frequency and distribution of sow deaths across reproductive stages.

Consistent with these physiological differences, sow mortality shows stage-dependent variation. However, most existing studies have focused on counts or proportions of sow deaths, rather than on incidence rates that account for the duration of exposure within each reproductive stage [12, 19, 32, 33]. The contribution of reproductive stages to sow mortality risk has not been fully quantified. Similarly, the influence of sow-level and herd-level factors on mortality across reproductive stages, when assessed using incidence rates rather than absolute counts, has not been systematically investigated. Filling this gap could provide more precise identification of sows at higher risk of death and inform targeted management strategies.

In commercial pig production, data management and recording systems are essential for monitoring productivity and health. However, many farms still rely on manual record-keeping, resulting in inconsistent or incomplete data. Studies have shown wide variation in data quality across farms depending on herd size, management software, and personnel training [34, 35]. Moreover, in most cases, these records are mainly used for routine purposes, such as generating sow cards, task schedules, or brief performance overviews, rather than being analyzed to extract meaningful insights [36]. When systematically analyzed, herd data can provide a powerful foundation for evidence-based management, enabling producers and veterinarians to enhance sow lifetime reproductive potential and overall production efficiency [37, 38]. Spain is one of the most important swine production countries in the world, with almost 35,000,00 pigs produced in 2023 [39]. However, studies over sow mortality are rare in this region [10, 40], particularly using large-scale data.

Therefore, given the observed stage-dependent variation in sow mortality, the limited availability of large-scale production data, and the scarcity of studies in Spain, this study aims to analyze sow mortality trends across reproductive stages in 16 Spanish swine farms over a six-year period and to identify key factors contributing to elevated mortality and individual sow mortality risk using production records.

## Methods

### Dataset preparation

The study included production records from sixteen commercial breeding farms located in northeastern Spain, including eight farms in Aragon, seven in Catalonia, and one in Navarre. These records were collected daily as part of the farms’ routine management procedures. All participating farms belonged to the same production company and used a standardized electronic recording system to collect production and sow removal data. These farms were chosen based on the company’s prior experience, as they provided more detailed and consistent classification of sow mortality compared to the rest of the farms in the system (Antonio Martinez; personal communication, 2024).

Data management, descriptive statistics and analyses were performed using R studio [41].

The dataset comprised 488,849 service records from 106,351 sows between January 2019 and December 2024. Gilts that were removed before their first service were excluded from the analysis, since not all farms consistently recorded such removals. The dataset used for this study was composed of four files from the production record system with sow individual records. Variables used from each file are summarized in Table 1.

**Table 1:** Summary of the files and fields used to create the data frames analyzed in this study

Files	Fields
FARM CENSUS	Sow ID, Entering Date, Parity at Farrowing, Farrowing Date, Elimination Date, Elimination Type (Death, Euthanasia or Culling), Cause of Elimination, Breed, Non-Productive Days at Elimination.
LITTERS BORN	Sow ID, Parity, Farrowing Date, Total Born Piglets, Live Born Piglets, Dead Born Piglets, Mummies, Adopted Weaned, Weaned Piglets, Breed.
SERVICE INFORMATION	Service Date, Sow ID, Parity, Service Number, Previous Service Date.
SERVICE OF ENTERED SOWS	Service Date, Result, Type, Sow ID, Boar ID, Farrowing Date, Entry Date, Age at First Service.

Based on established criteria from previous studies, certain variable values were identified as extreme and treated as missing data (the value was replaced by NA, i.e., “not available”, and not taken account in the analysis), while the service records remained in the study for analyses. These included: gilt age at first service below 160 days or above 400 days (11,008 records; [42]); gestation lengths shorter than 105 days or longer than 125 days (73 records; [43]); total piglets born fewer than 1 or greater than 30 (173 records; [44]); values were excluded, such as cases where piglet deaths exceeded the total number of piglets born plus those fostered to the sow (1,486 records) and nonproductive sow days of 366 days or more (94 records). Weaned piglet counts for sows involved in cross-fostering (52,525 records) were excluded.

For each sow, reproductive records were organized into two periods based on major risk windows for mortality: the insemination-to-farrowing period (gestation) and the farrowing-to-next-insemination period (post-farrowing). Each period was divided into discrete intervals of fixed length (14 days for gestation; 7 days for post-farrowing). For each interval, the sow was considered at risk unless a terminating event occurred (farrowing, next service, removal, or the end of the study period), in which case the risk time was truncated to the actual number of days observed.

Sow-years at risk were calculated by summing risk time across all intervals for each sow, divided by 365.25:

$$\:{t}_{i,v}=\left\{\begin{array}{c}{L}_{q},v<{K}_{i}-1\\\:{\varDelta\:}_{i,v},v={K}_{i}-1\end{array}\right.$$

where *t*_*i, v*_ is the risk time contributed by sow *i* in interval *v*, *K*_*i*_ is the total number of intervals for sow *i*, *L*_*q*_ is the fixed length (in days) of each interval under reproductive period *q* (*q = G* for the insemination-to-farrowing period, with *L*_*G*_ = 14 days; *q = F* for the farrowing-to-next-insemination period, with *L*_*F*_ = 7 days), and *Δ*_*i, v*_ is the observed number of days contributed in the final interval, calculated from the first day of the final interval to the terminating event (farrowing, next service, removal, or the end of follow-up), inclusive of both dates.

The individual risk time contribution *t*_*i, v*_ was aggregated to calculate sow-years at the relevant analytical stratum in the later analytics:

$$\:SY_A=\frac{\left[{\sum}_{i}{\sum_{v\in A}}t_{i,v}\right]}{365.25}$$

where *SY*_*A*_ is the sow-years at risk for a specified analytical stratum *A*, calculated as the total contributed risk time across all sow-interval observations included in that stratum divided by 365.25 days. The stratum *A* may represent the whole farm population or any predefined subgroup (e.g., farm, reproductive period, month, parity group, or their combinations).

### Definitions

In this study, a sow is defined as a female pig that had been inseminated at least once. Parity was defined as the number of farrowing completed, meaning that parity increased only after successful farrowing. A service is defined as any insemination event occurring during estrus and could include one or multiple mating events within the same estrus cycle.

Sows can be eliminated by culling, death or euthanasia. Deaths were classified into the following major categories, based on their frequency of occurrence: accident, illness, peripartum, prolapse, sudden death, ulcer, locomotor problems, clostridium, moribund, and others. All remaining infrequent or insufficiently specified causes were grouped into an “others” category. The definitions of each mortality category were provided by the company, and farm personnel classified each death according to these standardized guidelines. The definitions of the categories are as follows:

- Accident: deaths resulting from trauma or physical injury, such as crushing, falls, or entrapment within housing facilities.
- Illness: deaths caused by clinically diagnosed diseases not falling into other specific categories, including systemic infections or chronic conditions.
- Peripartum: deaths occurring around the time of farrowing, often associated with complications of parturition or immediate postpartum physiological stress.
- Prolapse: deaths resulting from vaginal, uterine, or rectal prolapse, typically occurring during late gestation or shortly after farrowing.
- Sudden death: deaths without prior clinical signs, characterized by abrupt onset and rapid progression.
- Ulcer: deaths associated with gastric or gastric-proximal intestinal ulceration leading to hemorrhage or perforation.
- Locomotor problems: deaths attributable to lameness, fractures, arthritis, or other musculoskeletal disorders impairing mobility.
- Clostridium: deaths with compatible clinical signs of infection with clostridium species (e.g. bloated).
- Moribund: Terminal ill or with hopeless prognosis animals that were identified to be euthanized but died before euthanasia.

Seasons were defined according to conventional meteorological periods [45]: Winter (from December 1st to February 28/29th ), Spring (from March 1st to May 31st ), Summer (from June 1st to August 31st ) and Fall (from September 1st to November 30th ).

The risk factors analyzed in this study were derived from four levels: herd-level, sow-level, parity-level, and service-level data.

At the herd level, there variables were assessed:

- Herd size: the average number of sows present on the farm per day, averaged annually and then across the six-year study period (2019–2024).
- Piglets weaned per sow per year (PWSY): the farm-level average number of piglets weaned per sow per year, averaged across the same six-year period.
- Whether the farm bred their own gilts.

At the sow level, the following variables were assessed:

- Parity at removal: the parity number at which a sow was removed from the herd (through death, euthanasia, or culling).
- Non-productive sow days: a database-derived variable representing the cumulative number of days a sow was classified as nonproductive during her time on the farm.
- Age at first service: the sow’s age, in days, at the time of her first recorded service when she entered the farm.
- Total piglets born alive per sow: the cumulative number of piglets born alive across all parities recorded for a sow during the entire study period.
- Total piglets weaned per sow: the cumulative number of piglets weaned across all parities recorded for a sow during the entire study period.
- Days from last farrowing to death: the number of days between a sow’s most recent farrowing and her death.
- Days from last service to death: the number of days between a sow’s most recent service and her death.

At the parity level, the following variables were assessed:

- Number of parities at service: the parity number of the sow at the time a given service was recorded (i.e., the number of parities the sow had already completed prior to that service).
- Gestational length: the number of days between service (the last service event, if the sow received service attempts for a given parity) and farrowing for that parity.
- Piglets born alive: the number of piglets born alive in a given parity.
- Piglets born still: the number of stillborn piglets in a given parity.
- Piglets born mummified: the number of mummified fetuses in a given parity.
- Piglets weaned: the number of piglets weaned in a given parity.

At the service level, one variable was assessed:

- Number of services: the total number of service attempts recorded for a sow within a given reproductive cycle prior to a successful conception.

### Descriptive analysis

A descriptive analysis was conducted to characterize sow mortality patterns and herd-, sow-, parity-, and service-level variables across the study population. These included annual mortality rates, distributions of causes of death, weekly mortality trends, risk of death according to parity at removal, the temporal distribution of sow deaths across the reproductive cycle, and mortality incidence across different stages of gestation. Continuous variables were summarized using the mean, standard deviation (SD), median, interquartile range (IQR), and range to describe their central tendency and variability.

For the overall weekly mortality analysis, death counts and average weekly sow population were aggregated by week, and weekly mortality was calculated as the ratio of deaths to population.

$$\:Weekly\:mortality\:\left(\%\right)=\frac{{D}_{w}}{\left(\frac{\sum_{d=1}^{7}{N}_{d}}{7}\right)}\times\:\:100$$

where *D*_*w*_ is the number of sow deaths during week *w*, and *N*_*d*_ is the sow population on day *d* of that week.

The risk of death of sows was calculated as the number of dead sows divided by the number of removed sows, multiplied by 100.

$$\:Risk\:of\:death\:\left(\%\right)=\frac{D}{R}\times\:\:100$$

where *D* is the number of dead sows and *R* is the number of removed sows (including deaths, euthanasia, and culling).

Weekly mortality incidence of sow in different gestation month were analyzed using recorded sow deaths and sow population data from 2019 to 2024. Each sow was assigned a pregnancy month based on the time elapsed from service, and weekly death counts and sow-years at risk were aggregated by week and pregnancy month. Mortality incidence per week was calculated as the number of deaths divided by the sow-years at risk.

$$\:{Mortality\:incidence}_{w,m}\:=\:\frac{{D}_{w,m}}{{SY}_{w,m}}\times\:\:1000$$

where *w* denotes the calendar week during the study period (2019–2024), *m* denotes the pregnancy month (months 1–4 of the gestation).*D*_*w, m*_ is the number of sow deaths during week *w* among sows contributing risk time within pregnancy month *m*, and *SY*_*w, m*_ is *SY*_*A*_ with the analytical stratum *A* defined as calendar week *w* and pregnancy month *m*.

### Incidence rate analysis

Using the GLIMMIX procedure, two-level Poisson regression models were applied:

1. To estimate the incidence rate of death while accounting for herd variability and to analyse its associations with herd-level factors.

The herd-level factors that we examined were herd size, PWSY, and whether the farm bred their own gilts. Herd size was calculated annually for each farm as the average number of sows present on the farm per day during that year, based on each sow’s daily presence. Farm-level herd size was then obtained by averaging the annual values across the six years of the study period (2019–2024).

$$\begin{array}{c}\\\:{Days}_{i,y}={End\:date}_{i,y}-{Start\:date}_{i,y}+1\\\:\\{Herd\:size}_{y}=\frac{\varSigma\:{Days}_{i,y}}{365.25}\\\:\\{\overline{Herd\:size}}_{farm}=\frac{{\sum\:}_{y=2019}^{2024}Herd\;size_{y}}{6}\end{array}$$

where *Start date*_*i, y*_ is the later of sow *i*’s entry date and the first day of calendar year *y*; *End date*_*i, y*_ is the earlier of sow *i*’s removal date and the last day of calendar year *y*; *Days*_*i, y*_ is the number of days sow *i* was present on the farm during calendar year *y* (as defined above); *Herd size*_*y*_ is the average annual herd size (in sow-years) for calendar year *y*; and ${\overline{Herd\:size}}_{farm}$ is the farm-level average herd size across the six-year study period.

PWSY was extracted annually from the farm database for 2019–2024 and averaged across the six years to obtain a single farm-level value.

$$\:{\overline{PWSY}}_{farm}=\frac{{\sum\:}_{y=2019}^{2024}PWS{Y}_{y}}{6}$$

where *PWSY*_*y*_ is the farm-level PWSY value for year *y*, extracted directly from the database.

An intercept-only Poisson model with an offset and no fixed or random effects was applied to estimate the simple incidence rate, expressed per 1,000 sow-years at risk:

$$\begin{array}{c}\\\:\mathrm{log}\left({\mu\:}_{j}\right)={\beta\:}_{0}+\mathrm{log}\left(\frac{S{Y}_{j}}{1000}\right)\\\:IR={e}^{{\beta\:}_{0}}\end{array}$$

where *µ*_*j*_ is the expected number of dead sows for farm *j*, *β*_*0*_ is the intercept, and *SY*_*j*_ is the sow-years at risk for farm * j*, corresponding to the general definition of *SY*_*A*_ with analytical stratum *A = J*. The overall incidence rate (IR) is obtained by exponentiating the intercept.

A random intercept for herd was then added to estimate overall and herd-level incidence rates while accounting for herd effects, along with their 95% confidence intervals (CI):

$$\begin{array}{c}\:\mathrm{log}\left({\mu\:}_{j}\right)={\beta\:}_{0}+{u}_{j}+\mathrm{log}\left(\frac{S{Y}_{j}}{1000}\right)\\\:{u}_{j}\sim{N}\left(0,{\sigma\:}_{u}^{2}\right)\\\:I{R}_{j}={e}^{{\beta\:}_{0}+{u}_{j}}\end{array}$$

where the subscript *j* denotes farm, *u*_*j*_ is the herd-level random effect assumed to follow a normal distribution with mean 0 and variance $\:{\sigma\:}_{u}^{2}$; *SY*_*j, s*_ is the sow-years at risk for farm *j* within analytical stratum *s*, corresponding to the general definition of *SY*_*A*_ with analytical stratum *A = (j*,* s)*. and *IR*_*j*_ is the resulting herd-level incidence rate.

Whether the farm bred their own gilts (no vs. yes) modeled as a fixed effect (see equation in the next section), while numerical indicators such as herd size and PWSY evaluated using Pearson’s correlations with herd-level incidence rates.

2) To examine the associations between the incidence rate of death and parity-level factors.

Assessed parity-level factors included weeks from service or farrowing, parity at service or farrowing, re-service, and service or farrowing season. To address the second objective, these factors were added to the models as fixed effects in a univariate manner (i.e., one factor at a time). For analyses of time since service or farrowing, individual records were expanded into week-level risk sets, covering the period from each reproductive event (service or farrowing) until the next event or sow death (see in Data Preparation section); weekly mortality outcomes and sow-years at risk were calculated for each interval as described in the Data Preparation section (*SY*_*j, s*_, with *s* denoting the stratification variable of interest).

Each parity-level factor was modeled univariately as a fixed effect, together with a random intercept for farm:

$$\begin{array}{c}\:\mathrm{log}\left({\mu\:}_{j,s}\right)={\beta\:}_{0}+{\beta\:}_{1}{X}_{s}+{u}_{j}+\mathrm{log}\left(\frac{S{Y}_{j,s}}{1000}\right)\\\:{u}_{j}\sim{N}\left(0,{\sigma\:}_{u}^{2}\right)\end{array}$$

where *X*_*s*_ denotes the parity-level factor being assessed (e.g., week since service/farrowing, parity, repeat services, or season) within stratum *s*, *β*_*1*_ is the corresponding fixed-effect coefficient, and *SY*_*j, s*_ is the sow-years at risk for farm *j* within stratum *s*, as defined previously. Each factor was assessed in a separate model.

### Matched case-control study

Three matched studies were performed to investigate differences between dead sows (cases) and their matched controls, defined as sows that left the herd for reasons other than death or that remained alive:

1. Age at first service was compared between gilts that died before their first parity and their matched controls (gilts that did give birth to the first parity). Controls were matched on parity, farm, number of services, year of service, and season.
2. Litter reproductive performance was compared between case parity records (last parities before sow death) and matched control parity records from sows that did not die after the corresponding parity. Matching was based on parity number, farm, farrowing year, and season. Variables included gestation length, total piglets born, stillborn piglets, mummified fetuses, and piglets weaned. This analysis aimed to identify potential pre-death signals in litter performance.
3. Lifetime performance metrics were evaluated by comparing deceased sows with their matched controls (those alive or removed for reasons other than death) to assess differences in long-term productivity. For each sow, average lifetime litter performance was calculated by averaging the corresponding variable across all of the sow’s parities:

$$\:{\overline{X}}_{i}=\frac{{\sum\:}_{p=1}^{{P}_{i}}{X}_{i,p}}{{P}_{i}}$$

where *X*_*i, p*_ is the value of the variable of interest (e.g., piglets weaned) for sow *i* at parity *p*, *P*_*i*_ is the total number of parities recorded for sow *i*, and *X̄*_*i*_ is the resulting lifetime average for sow *i*. Controls were matched with cases based on farm, first service year, and season.

The R package “ccoptimalmatch” was used in this study to match each case with up to four controls. Although not all cases could be matched with four controls, the majority were successfully matched at this 1:4 ratio, which has been shown to maximize cost-effectiveness and statistical efficiency [46].

For each variable assessed in the three analyses described above, case records and all corresponding matched-control records were treated as two independent samples and compared using the Wilcoxon rank-sum test. This analysis provided an overall comparison of the distributions of case and control values.

$$\:W=\sum\limits_{c=1}^{{n}_{1}}{R}_{c}-\frac{{n}_{1}\left({n}_{1}+1\right)}{2}$$

where *R*_*c*_ is the rank of the *c*-th case observation in the combined ranking of case and control values, and *n*_*1*_ is the number of case records.

To compare the variable within the matched-set structure, a stratified Wilcoxon rank-sum test (van Elteren test) was subsequently applied to the same case and control records, stratified by matched set, using the “coin” package in R:

$\:T=\frac{{\sum\:}_{k=1}^{K}{w}_{k}\left({S}_{k}-E\left({S}_{k}\right)\right)}{\sqrt{{\sum\:}_{k=1}^{K}{w}_{k}^{2}Var\left({S}_{k}\right)}}$where *k* denotes the matched set, *S*_*k*_ is the Wilcoxon rank-sum statistic computed within matched set k, *E(S*_*k*_*)* and *Var(S*_*k*_*)* are the expected value and variance of *S*_*k*_ under the null hypothesis of no difference between cases and controls, *w*_*k*_ is the weight assigned to matched set *k*, and *K* is the total number of matched sets. Under the null hypothesis, *T* asymptotically follows a standard normal distribution.

The p-values from both tests were reported. The Wilcoxon rank-sum test provided an overall comparison, whereas the van Elteren test accounted for the matched-set structure. Concordant results from the two analyses were interpreted as supporting the robustness of the findings.

## Results

The dataset from the 16 herds comprised 488,849 service records from 106,351 sows. Out of 106,351 sows, 76,607 sows were removed during the study period. The removal reasons were culling (71.6%; 54,848 sows), dead (21.7%; 16,634 sows), euthanasia (6.4%; 4,877 sows), and others (0.3%; 248 sows).

**Fig. 1:**
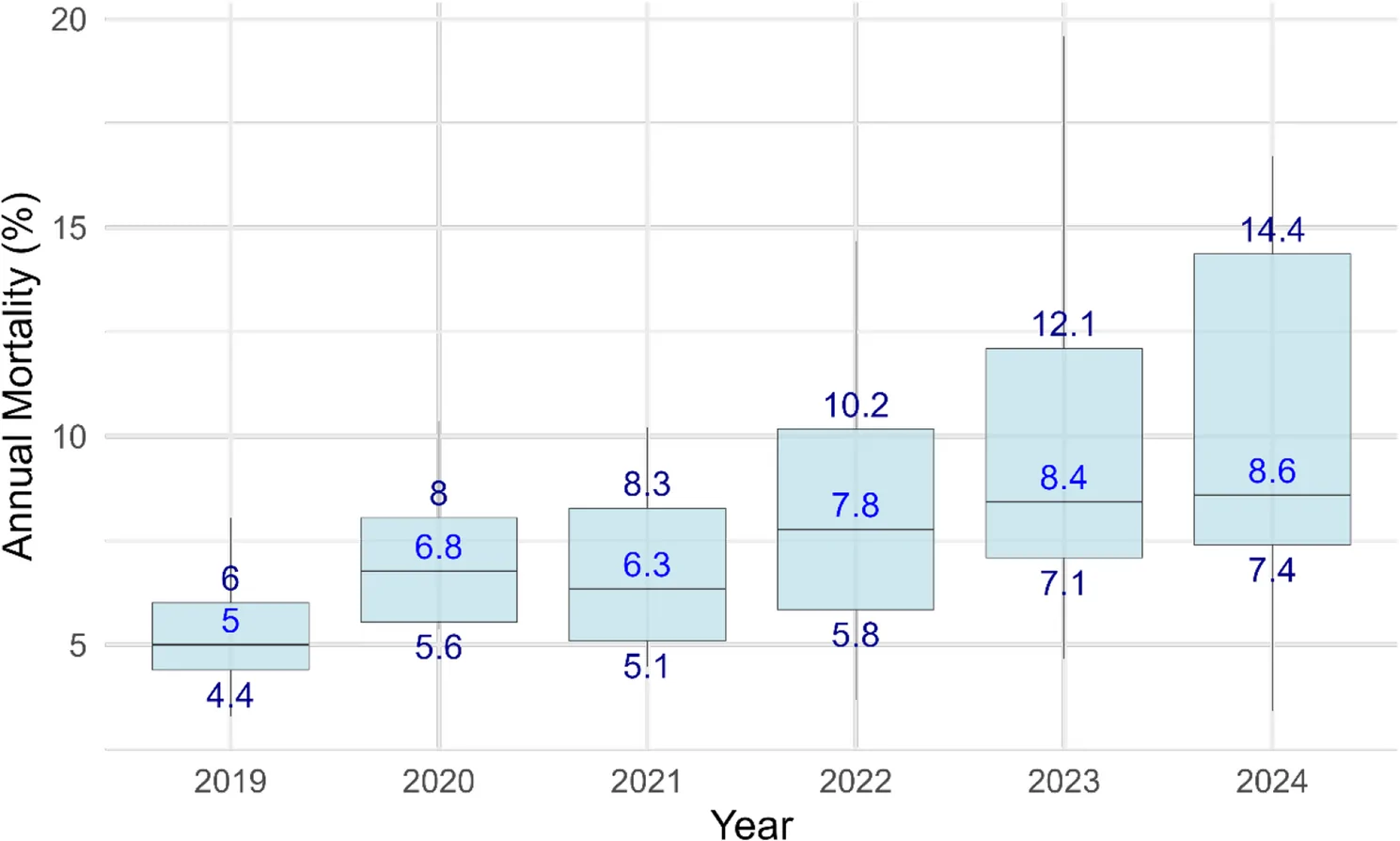
Boxplot of annual mortality rates across 16 farms from 2019–2024. The values at the top, middle, and bottom of each box indicate the 75th percentile, median (50th percentile), and 25th percentile, respectively

The average annual mortality rate for the 16 farms was 5.20% (95%CI : 4.45% − 5.95%), 7.06% (95%CI : 6.11% − 8.00%), 6.80% (95%CI : 5.76% − 7.83%), 8.15% (95%CI : 6.58% − 9.71%), 9.82% (95%CI : 7.73% − 11.91%), 9.74% (95%CI : 7.42% − 12.06%) from 2019 to 2024, respectively. A summary of each farm yearly mortality can be found in Fig. 1.

**Fig. 2:**
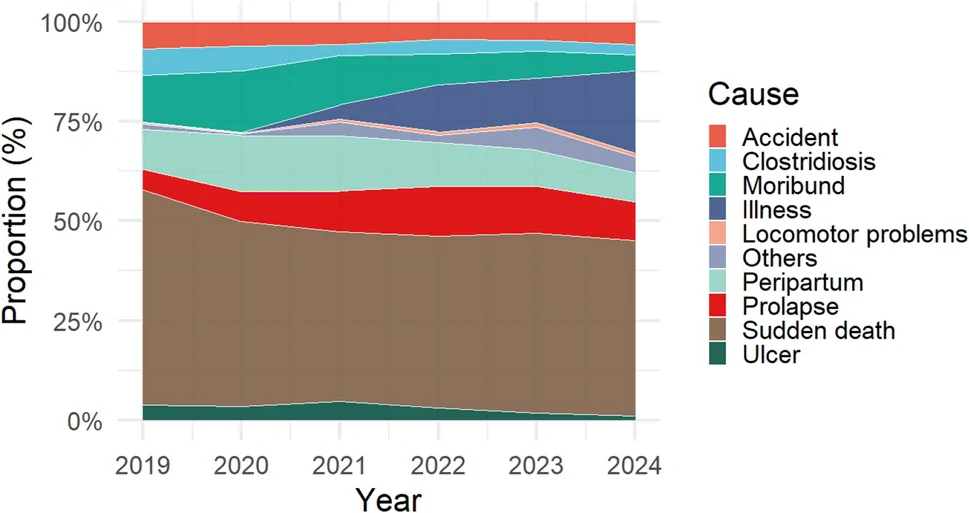
Cumulative annual distribution of the top 9 causes of death and other causes (2019–2024). Each colored area corresponds to a cause, showing its proportion in total deaths over the years. Colors distinguish causes

The overall causes of mortality (dead) during the studied period were accident (5.42%; 901 cases), clostridium (3.75%; 624 cases), moribund (8.73%; 1453 cases), illness (9.70%; 1613 cases), locomotor problems (0.73%; 122 cases), peripartum (10.45%; 1738 cases), prolapse (10.00%; 1664 cases), sudden death (45.17%; 7514), ulcer (2.71%; 451 cases), or others (2.94%; 489 cases). The distribution of death causes from each year is shown in Fig. 2.

**Fig. 3:**
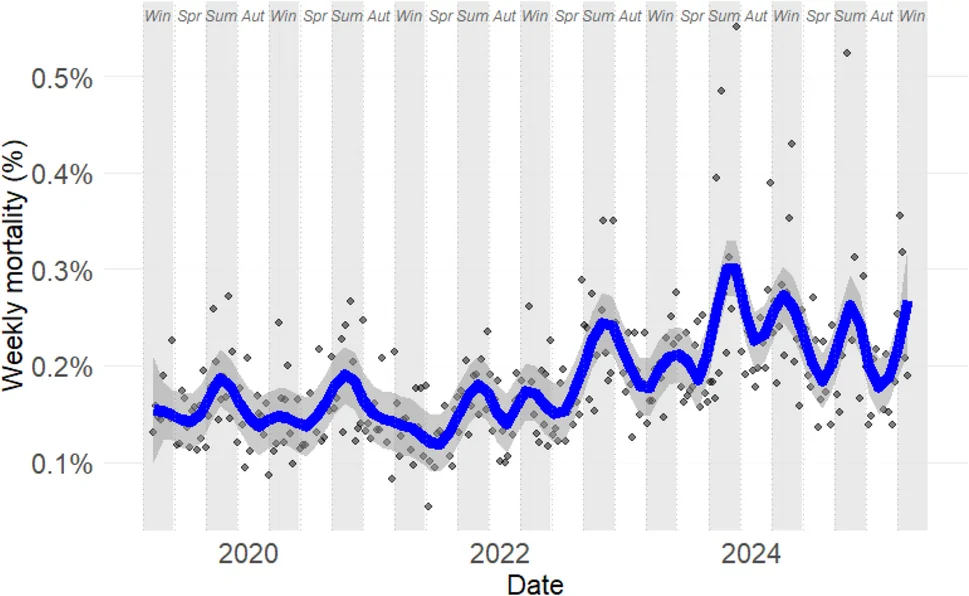
Percentage of calculated sow weekly mortality (blue line) and its 95% CI (grey area) during the period of study (January 1st, 2019, to December 31st, 2024). Seasonal periods were shown as shaded and labeled background bands for comparison (winter: Win, spring: Spr, summer: Sum, autumn: Aut) and individual weekly mortality points were plotted. X-axis labels were formatted by date, with major breaks every six months and minor breaks every month

As shown in Fig. 3, weekly mortality rates exhibited an overall upward trend, especially after 2022, with noticeable seasonal peaks during the summer and winter months.

The descriptive statistics for the different factors used for the farm- and sow-level analysis are presented in Table 2. The average herd size and PWSY during the period 2019–2024 were 1998.89 sows and 23.73, respectively.

**Table 2:** Descriptive statistics of herd and sow-level factors used for the analysis of the 16 selected farms. SD: Standard deviation, IQR: Interquartile Range between 25th and 75th percentiles

Measurements	*N*	Mean	SD	Median	IQR	Range
Herd level data						
Herd size	16	1998.89	2005.78	1288.8	(379.68 − 2197.91)	452.74 − 8749.46
PWSY	16	23.73	2.05	23.71	(22.51 − 24.92)	21.28 − 29.25
Sow-level data						
Parity at removal	106,351	4.31	3.11	4	(1.5 − 6.5)	0 − 15
Nonproductive sow days	76,587	45.34	50.29	26	(-6.5 − 58.5)	0 − 359
Age at first service, days old	103,403	275.6	37.09	270	(245.5 − 294.5)	160 − 401
Total piglets born alive per sow	88,954	58.59	38.77	51	(21.5 − 80.5)	0 − 278
Total piglets weaned per sow	88,954	49.03	33.21	43	(19 − 67)	0 − 283
Days from last farrowing to death	7387	16.62	19.75	13	(3.5 − 22.5)	0 − 529
Days from last service to death	9247	78.73	38.88	92	(58.5 − 125.5)	0 − 777
Parity-level data						
Number of parities at service	106,442	3.87	3	3	(0.5 − 5.5)	0 − 16
Gestational length	380,527	115.32	1.4	115	(114.5 − 115.5)	105 − 125
Piglets born alive	344,745	13.94	3.91	14	(12 − 16)	0 − 30
Piglets born still	344,745	1.33	1.87	1	(0 − 2)	0 − 27
Piglets born mummified	344,745	0.51	1.24	0	(-0.5 − 0.5)	0 − 25
Piglets weaned	291,635	11.93	2.59	12	(11 − 13)	1 − 30
Service-level data	291,635	11.93	2.59	12	(11 − 13)	1 − 30
Number of services	439,788	1.11	0.36	1	(1 − 1)	1 − 9

Table 3 details the risk and proportion of death removals across parities. 15.64% of records (16,634 out of 106,351) indicated removal due to death. The risk of death was highest in the parity 6 + group (5.9%), followed by parity 1 (3.1%). Gilts were the ones with the lowest death risk (1.6%). It increased to 3.1% at parity 1 and then steadily decreased with increasing parity, remaining relatively low through parity 5 (2.4%). However, a marked increase was observed in the parity 6 + group, where the risk rose sharply to 5.9%.

**Table 3:** Risk and proportion of death by parity at removal

Parity group	Number of dead sows	Sows at risk	Removed sows	Risk of removal due to death, %^1^	Proportion of death, %^2^
0	1700	106,351	5717	1.6	29.7
1	3123	100,634	10,555	3.1	29.6
2	2479	90,079	7805	2.8	31.8
3	2227	82,274	7456	2.7	29.9
4	1844	74,818	6841	2.5	27
5	1636	67,977	6775	2.4	24.1
6 or higher	3625	61,202	31,459	5.9	11.5
Total	16,634	106,351	76,608	15.64	21.71

^1^ Denominator was the number of sows at risk

^2^ Denominator was the number of removed sows

**Fig. 4:**
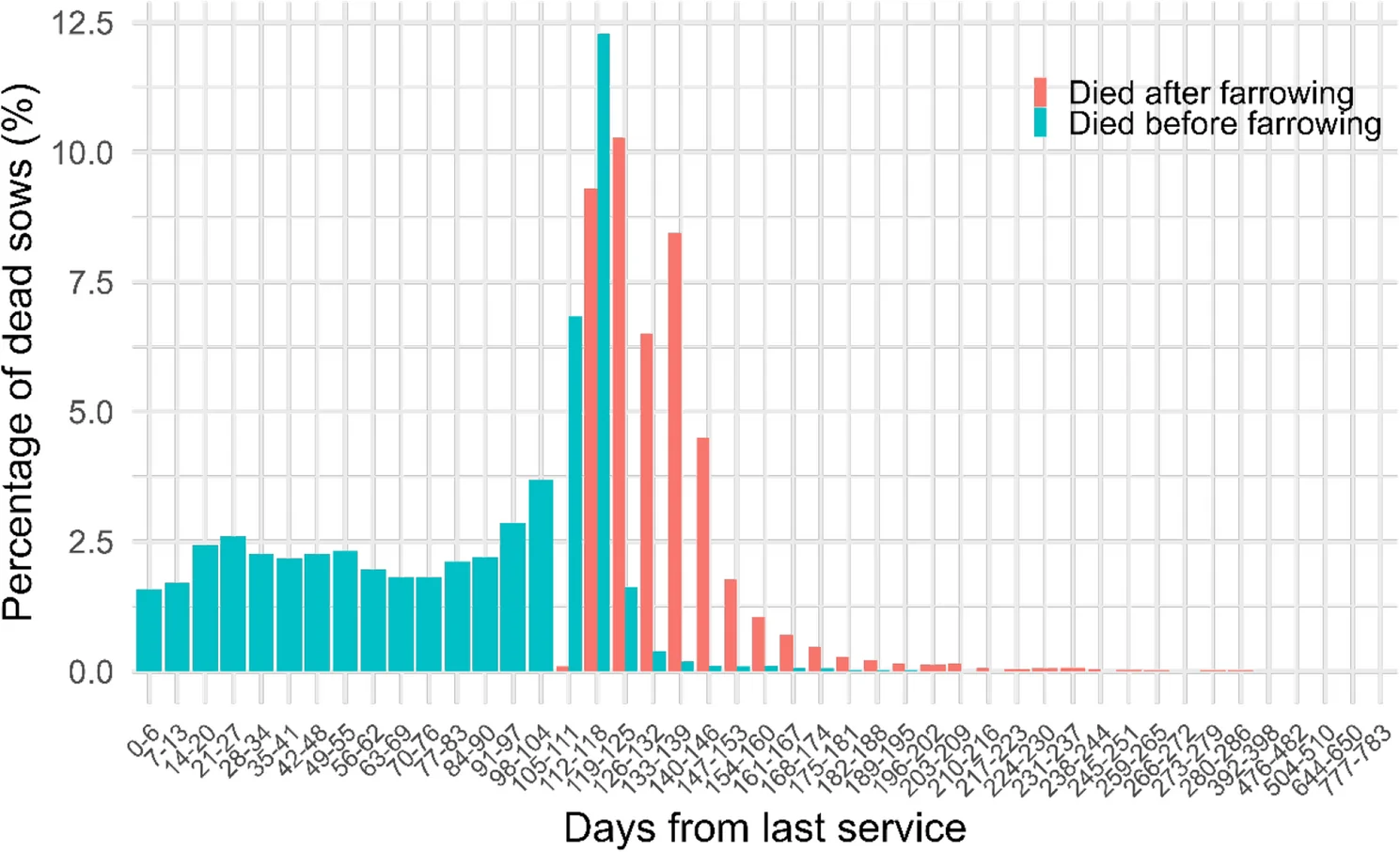
Relative mortality percentages from the total number of dead animals occurring at different times after the last recorded service. Blue: died before farrowing and therefore those animals do not have a registered farrowing event. Red: died after a registered farrowing event. Bins in both categories coincide have narrower bars

Among the total sow deaths (16,634), 44.41% (7,387) occurred after farrowing, while 55.59% (9,247) happened before farrowing (Fig. 4). The weeks with the highest mortality rates were as follows: 105–111 days of gestation, accounting for 6.93% (1,153/16,634) of deaths; 112–118 days, the peak period, with 21.57% (3,588/16,634); 119–125 days at 11.91% (1,981/16,634); 126–132 days at 6.89% (1,136/16,634); and 133–139 days at 8.63% (1,435/16,634).

**Fig. 5:**
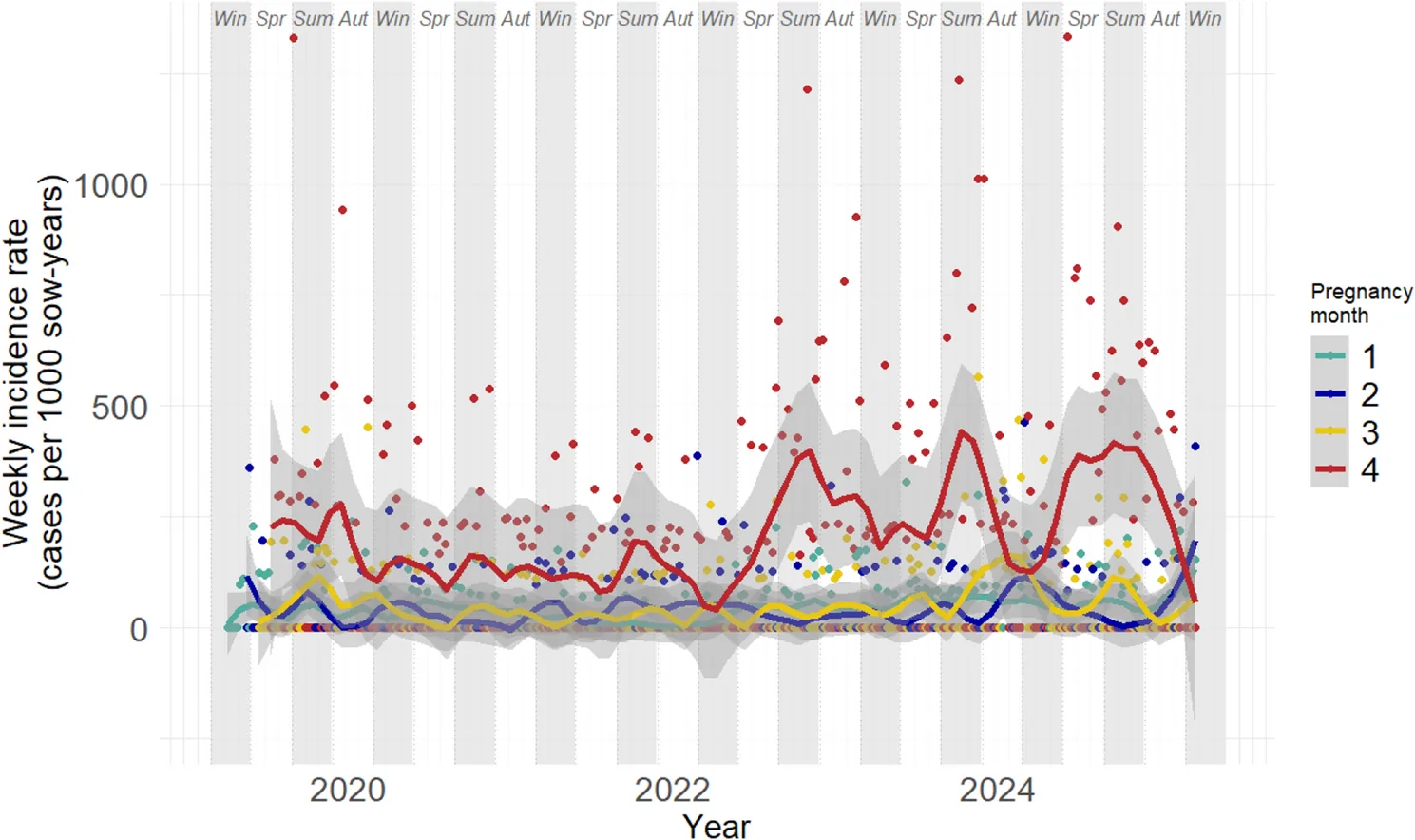
Weekly mortality incidence rate (cases per 1,000 sow-years) of pigs in each month of the pregnant period (green = month 1, blue = month 2, yellow = month 3, red = month 4) with fitted trend lines and 95% CI. Colored dots represent weekly mortality incidence rate, calculated with death count of the week divided by the sow-years at risk of the week (the accumulated time at risk contributed by all sows), while solid lines show smoothed trends. Seasonal periods were shown as shaded and labeled background bands for comparison (winter: Win, spring: Spr, summer: Sum, autumn: Aut). Grey-shaded areas represent 95% confidence intervals for the fitted trends

The highest mortality incidence was observed in pigs during their last month of pregnancy, with a pronounced peak occurring specifically in the summer (Fig. 5). The contrast between the last month of pregnancy and the other months, as well as the difference between summer and the other seasons, became more evident after 2022.

The overall sow mortality incidence rate across the 16 farms was 99.26 deaths per 1,000 sow-years (95% CI: 97.84-100.71), as shown in Table 4. The simple estimated mortality incidence rate for serviced sows was 67.44 (95% CI: 66.12–68.78), while farrowed sows had a much higher incidence of 242.5 (95% CI: 237.7-247.36) deaths per 1,000 sow-years. After taking herds effects into account, serviced animals had a median of 62.866 (95% CI: 56.04–70.520), whereas farrowed sows had a median of 193.831 (95% CI: 158.748-236.668) deaths per 1,000 sow-years (Table 4).

**Table 4:** Incidence rates of sow mortality by reproductive period (service to farrowing, farrowing to subsequent service, and total period), estimated using Poisson random-intercept models with an offset for log(sow-years at risk/1000), with comparison by replacement gilt source and correlation with herd management measurements

Measurements	Period		
	From service until farrowing	From farrowing until subsequent service	Total period
Number of cases^1^	9247	7387	16,634
Number of sows at risk^2^	106,351	95,255	106,351
Sow-years at risk	137112.69	30461.33	167574.02
Incidence rate cases per 1000 sow-years (95% CI)^3^			
Simple estimate (95% CI)	67.44 (66.12, 68.78)	242.5 (237.7, 247.36)	99.26 (97.84, 100.71)
Estimate taking herd effect into account (95% CI)	62.866 (56.043, 70.520)	193.83 (158.75, 236.67)	87.795 (77.11, 99.96)
Model intercept (SE)	4.141 (0.06)	5.267 (0.10)	4.475 (0.07)
Random herd effect (SD)	0.0515 (0.23)	0.1603 (0.40)	0.0681 (0.26)
Incidence rate (purchased / internal replacement gilts) (*p*-value)^4^	60.01 / 76.48 (0.07)	175.16 / 299.87(0.01)	82.11 / 117.06 (0.01)
Pearson’s correlation coefficient with herd management measurements (*p*-value)			
Herd size	0.23 (0.40)	0.44 (0.09)	0.36 (0.17)
PWSY	0.24 (0.37)	0.40 (0.12)	0.37 (0.15)

^1^ Number of sow deaths during the specific period

^2^ Number of sows contributing risk time during the specific period

^3^ The Simple estimate represents the overall sow-level incidence rate. The herd-adjusted estimate represents the median herd-level incidence rate estimated from the random-intercept model accounting for herd effects

^4^ Incidence rates were estimated from a Poisson random-intercept model with offset and herd as the random effect and replacement gilt source (purchased vs. internal) as a fixed effect. *P*-values reflect the comparison between the two groups

To further explore herd-level factors, a fixed factor was included in the model to assess the effect of self-breeding of gilts. Results showed that farms that bred their own gilts had a significantly higher mortality incidence in farrowed sows (p-value = 0.01) and observation in total period (p-value = 0.01). Additionally, Pearson’s correlation analysis, presented in Table 4, herd size and PWSY did not show a significant association with mortality incidence rates at any stage.

Death incidence in serviced sows was associated with the weeks from the last service, number of parities, number of services and service season (Table 5). There is a 3.69-fold increase at 14–15 weeks (101.085 cases per 1,000 sow-years), a 20.65-fold increase at 16–17 weeks (565.35 cases per 1,000 sow-years) and a 8.66-fold increase at 18 weeks and more, compared to the baseline incidence observed at 0–1 weeks (27.372 cases per 1,000 sow-years). The mortality incidence rate initially decreases with increasing parity but then rises again. Individuals at parity 2 exhibited the lowest mortality incidence. The highest incidence rates are observed at parity 5 (71.969 cases per 1,000 sow-years) and parity 6 or higher (68.516 cases per 1,000 sow-years), where they reach 1.22 and 1.16 times the incidence rate of parity 0 (59.049 cases per 1,000 sow-years), respectively. Sows that underwent repeated services had a pre-farrowing mortality incidence rate (55.81 cases per 1,000 sow-years) that was 0.87 times that of sows serviced only once (63.95 cases per 1,000 sow-years). The mortality incidence rate for sows serviced in the spring was the highest compared to other seasons, at 73.66 cases per 1,000 sow-years.

**Table 5:** Estimated incidence rate (cases per 1,000 sow-years) for death of serviced sows prior to farrowing, based on cohort data, estimated from a Poisson random-intercept model (offset: log[sow-years at risk/1000]) with herd as the random effect and weeks from service, number of parities at the service, number of services and service seasons as fixed effects

Variable^1^	Number of cases	Number of records	Total sow-years at risk	Incidence rate (95% CI)
Overall	9247	488,849	137112.68	62.87 (56.04, 70.52)
Weeks from service, p-value < 0.01				
0–1 (0–13 days)	546	488,849	18658.69	27.37 (23.89, 31.36)^a^
2–3 (14–27 days)	839	488,047	18166.41	43.21 (38.80, 48.13)^c^
4–5 (28–41 days)	738	484,671	17082.74	40.44 (36.20, 45.17)^bc^
6–7 (42–55 days)	761	476,567	16454.39	43.32 (38.81, 48.35)^c^
8–9 (56–69 days)	625	470,119	16061.84	36.47 (32.51, 40.91)^b^
10–11 (70–83 days)	655	465,355	15770.48	38.94 (34.76, 43.63)^bc^
12–13 (84–97 days)	841	461,607	15523.83	50.80 (45.61, 56.58)^d^
14–15 (98–111 days)	1750	458,368	15263.98	107.53 (97.68, 118.37)^e^
16–17 (112–125 days)	2313	454,331	3826.07	565.35 (515.00, 620.63)^f^
18 or more (over 126 days)	179	444,153	304.25	544.33 (459.50, 644.81)^f^
Number of parities at the service, p-value < 0.01				
0	1700	95,148	26722.52	59.05 (52.19, 66.81)^ab^
1	1403	83,982	23220.89	56.31 (52.46, 60.44)^a^
2	1254	70,510	19796.98	59.19 (55.02, 63.67)^abc^
3	1184	59,736	16953.68	65.41 (60.74, 70.46)^bcd^
4	1017	50,696	14368.94	66.43 (61.46, 71.80)^cd^
5	916	42,394	11950.16	71.97 (66.41, 77.99)^d^
6 or higher	1773	33,893	24099.51	68.52 (64.10, 73.24)^d^
Number of services, p-value < 0.01				
One service	8127	396,687	118800.16	63.95 (56.93, 71.83)^a^
Re-service	1120	92,162	18312.53	55.81 (48.9, 63.7)^b^
Service season, p-value < 0.01				
Spring	2800	121,650	35454.87	73.66 (64.53, 84.07)^c^
Summer	2146	122,176	34860.53	57.44 (50.25, 65.67)^a^
Autumn	2306	125,097	33967.58	63.26 (56.1, 71.34)^b^
Winter	1995	119,926	32829.71	56.67 (49.55, 64.82)^a^

*CI* Confidence interval

^1^ The variables were analyzed univariately in the model

^a−f^ Estimates within a group with different letters are different (*P* < 0.05)

Table 6, similar to Table 5, presents the associations between fixed factors and mortality incidence in farrowed sows, specifically examining the effects of weeks from farrowing, parity number, service times and season. The incidence of sow mortality was highest during the first week after farrowing (week 0), reaching 278.72 cases per 1,000 sow-years. This rate declined in the subsequent weeks but began to rise again after week 2. Notably, from week 7 onward, the mortality incidence exceeded that of the immediate post-farrowing period, with weekly incidence rates of 393.80, 323.90, 354.53, and 455.41 cases per 1,000 sow-years recorded during weeks 7 to 10 and beyond. Except for parity 1 (212.27 cases per 1,000 sow-years), mortality incidence in all other parities remained below the overall incidence rate (193.83 cases per 1,000 sow-years). The difference in mortality incidence between first-service and re-serviced sows is statistically significant (*p* < 0.01), indicating a higher mortality incidence rate in re-serviced sows compared to those first-serviced. Seasonal effects were evident, with the highest mortality incidence recorded in sows that farrowed during the summer (225.41 cases per 1,000 sow-years).

**Table 6:** Estimated incidence rate (cases per 1,000 sow-years) for dead sows after farrowing without subsequent service, using the cohort data, estimated from a Poisson random-intercept model (offset: log[sow-years at risk/1000]) with herd as the random effect and weeks from farrowing, number of parities at farrowing, number of services and farrowing seasons as fixed effects

Variable^1^	Number of cases	Number of records	Total sow-years at risk	Incidence rate (95% CI)
Overall	7387	488,849	30461.33	193.83 (158.75, 236.67)
Weeks from farrowing, p-value < 0.01				
0 (0–6 days)	2537	488,849	7238.93	278.72 (228.37, 340.18)^e^
1 (7–13 days)	1326	485,225	7160.67	147.32 (119.41, 181.76)^b^
2 (14–20 days)	1130	482,622	7066.13	127.23 (103.01, 157.16)^a^
3 (21–27 days)	1218	479,111	5326.58	182.22 (147.61, 224.95)^c^
4 (28–34 days)	452	471,135	1797.63	206.06 (164.83, 257.61)^cd^
5 (35–41 days)	240	462,405	735.39	263.92 (207.75, 335.29)^de^
6 (42–48 days)	126	488,104	425.86	240.53 (184, 314.44)^cde^
7 (49–55 days)	109	451,975	223.18	393.8 (298.59, 519.36)^f^
8 (56–62 days)	57	448,573	139.84	323.9 (232.91, 450.43)^def^
9 (63–69 days)	45	445,457	100.25	354.53 (248.34, 506.13)^ef^
10 or higher (over 70 days)	147	442,646	246.88	455.41 (351.14, 590.63)^f^
Number of parities at farrowing, *p*-value < 0.01				
1	1720	93,374	6406.61	212.27 (173.3, 260)^b^
2	1225	81,365	5110.02	192.16 (154.88, 238.4)^ab^
3	1043	68,812	4346.01	193.5 (155.76, 240.37)^ab^
4	827	58,740	3673.13	182.17 (146.32, 226.8)^a^
5	720	50,031	3105.56	187.53 (150.38, 233.84)^ab^
6 or higher	1852	41,427	7819.99	187.85 (151.78, 232.5)^a^
Number of services, *p*-value < 0.01				
One service	6633	396,687	27994.67	190 (155.77, 231.77)^a^
Re-service	754	92,162	2466.67	237.54 (192.05, 293.81)^b^
Farrowing season, *p*-value < 0.01				
Spring	1650	90,939	7355.37	180.03 (145.32, 223.04)^a^
Summer	2355	103,266	8326.77	225.41 (182.25, 278.8)^b^
Autumn	1789	100,064	8017.87	178.51 (145.64, 218.79)^a^
Winter	1593	86,331	6761.32	187.97 (151.7, 232.92)^a^

*CI* Confidence interval

^1^ The variables were analyzed univariately in the model

^a−f^ Estimates within a group with different letters are different (*P* < 0.05)

**Fig. 6:**
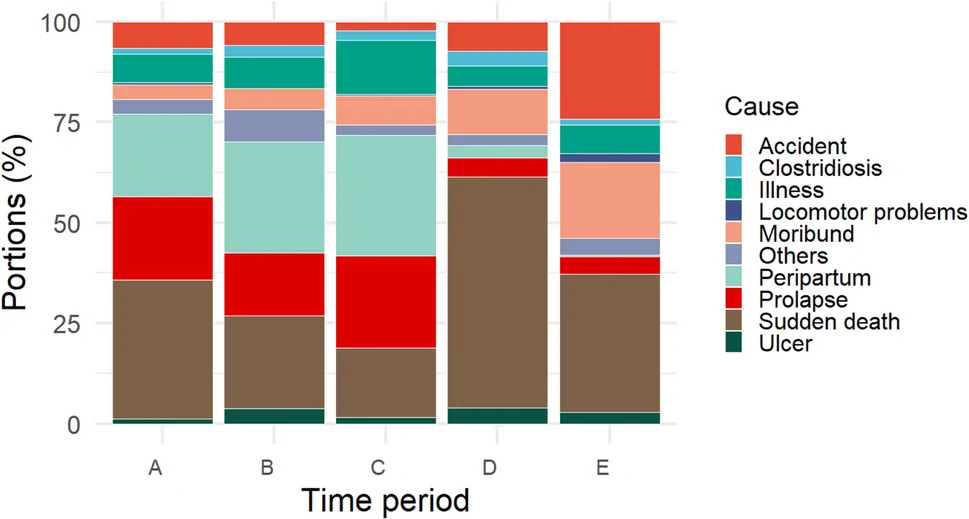
Proportion of death causes distribution of the critical periods of sow production cycle. Time period: A: 105–111 days after service, B: 112–118 days after service, C: 0–6 days after farrowing, D: 21–27 days after farrowing, E:7 weeks after farrowing. Only causes that account for more than 0.5% over the whole period are displayed, and within each year, only causes exceeding 0.5% are labeled. Different causes were in different colors and labeled with their portions in percentage

Based on the high-risk periods for sow mortality identified in this study, the causes of death were categorized and are presented in Fig. 6. Among sows that died between 105 and 111 days after service, sudden death was the most prevalent cause, followed by prolapse and peripartum. During 112–118 days after service, the most common cause became peripartum, followed by sudden death and prolapse. In the 0–6 days post-farrowing period, peripartum complications were still the leading cause, followed by prolapse, sudden death. For sows that died 21–27 days post-farrowing, sudden death was the most frequent cause, followed by moribund and accident. Lastly, 7 weeks after farrowing, the distribution shifted toward more accidents and moribund cases, while sudden death and still contributed to a measurable fraction of deaths.

Table 7 presents the comparisons of nine reproductive performance measurements between deceased sows and their matched controls. For the sows that died before giving birth for the first time, they had a significantly younger age at first service (*p* < 0.01). Among sows that died post-farrowing, their final parity was characterized by a shorter gestation length (*p* = 0.014), fewer total piglets born (*p* < 0.01), fewer live-born piglets (*p* < 0.01), and a higher incidence of stillbirths (*p* < 0.01). Stratified analyses further supported these associations. Although the overall number of piglets born mummified and weaned did not differ significantly between groups.

**Table 7:** Comparison of reproductive performance in the final reproductive cycle before death between case sows and matched control sows

Reproductive performance	Case		Control		*p*-value^7^
	Number of cases	Mean (SD)	Number of controls	Mean (SD)	
Gilt age at first service, days old^1^	1254	271.238 (35.845)	2646^3^	278.102 (33.618)	0.000 (0.000)
Gestational length, days^2^	7382	115.398 (0.877)	29,496^4^	115.457 (0.877)	0.014 (0.007)
Total piglets born^2^	6584	15.036 (4.704)	26,304^5^	15.848 (2.498)	0.000 (0.000)
Number of piglets born alive^2^	6584	12.447 (4.991)	26,304^5^	13.858 (2.364)	0.000 (0.000)
Number of piglets born still^2^	6584	2.000 (2.797)	26,304^5^	1.424 (1.077)	0.000 (0.000)
Number of piglets born mummified^2^	6584	0.589 (1.371)	26,304^5^	0.567 (0.727)	0.834 (0.828)
Number of piglets weaned^2^	2062	12.024 (3.440)	8230^6^	12.008 (1.845)	0.597 (0.559)

^1^ Cases (the first service records of sows that received the first service but died before they could give birth) and controls (the first service records of sows that didn’t die before their first parity) were matched by parity, farm, number of services received, service year and season

^2^ Cases (the last litter records of sows that died after the parity) and controls (the litter records of sows that didn’t die after the parity) were matched by parity, farm, number of services received, service year and season

^3^ 610 cases have 1 control, 216 cases have 2 controls, 108 cases have 3 controls, and 320 cases have 4 controls

^4^ 4 cases have 1 control, 4 cases have 2 controls, 12 cases have 3 controls, and 7362 cases have 4 controls

^5^ 4 cases have 1 control, 4 cases have 2 controls, 12 cases have 3 controls, and 6564 cases have 4 controls

^6^ 4 cases have 1 control, 2 cases have 2 controls, 2 cases have 3 controls, and 2054 cases have 4 controls

^7^ *p*-values for the stratified analyses are shown in the parentheses

Overall, deceased sows showed distinct lifetime performance patterns compared to their matched controls (Table 8; *p* < 0.01), including higher average numbers of total and live-born piglets per parity, but also increased stillbirths and mummified fetuses, but no significant difference in piglets weaned (*p* = 0.989). Additionally, deceased sows had a younger age at first service and fewer nonproductive days. Stratified analyses further supported these associations.

**Table 8:** Comparison of lifetime reproductive performance between case sows and matched control sows

Lifetime performance	Case^1^		Control^2^		*p*-value^8^
	Number of cases	Mean (SD)	Number of controls	Mean (SD)	
Gilt age at first service, days old	16,243	272.488 (35.589)	59,098^3^	274.136 (26.897)	0.000 (0.000)
Parity at removal	16,634	3.420 (2.582)	60,635^4^	5.043 (2.170)	0.000 (0.000)
Average number of piglets born per parity	12,943	14.398 (4.407)	47,219^5^	13.836 (4.226)	0.000 (0.000)
Average number of piglets born alive per parity	12,943	12.640 (4.196)	47,219^5^	12.192 (3.800)	0.000 (0.000)
Average number of piglets born still per parity	12,943	1.245 (1.471)	47,219^5^	1.162 (0.721)	0.000 (0.000)
Average number of piglets born mummified per parity	12,943	0.514 (0.983)	47,219^5^	0.482 (0.470)	0.000 (0.000)
Average number of piglets weaned per parity	11,117	9.611 (3.595)	41,323^6^	9.659 (3.406)	0.989 (0.189)
Nonproductive sow days	16,454	49.707 (54.031)	47,774^7^	51.119 (39.332)	0.000 (0.000)

^1^ Case sows were sows that died

^2^ Control sows were alive or removed for non-mortality reasons. The controls were matched with the case based on the farm, first service year and season

^3^ 73 cases have 1 control, 1,034 cases have 2 controls, 3,587 cases have 3 controls, and 11,549 cases have 4 controls

^4^ 68 cases have 1 control, 1,036 cases have 2 controls, 3,625 cases have 3 controls, and 11,905 cases have 4 controls

^5^ 10 cases have 1 control, 783 cases have 2 controls, 2,957 cases have 3 controls, and 9,193 cases have 4 controls

^6^ 352 cases have 2 controls, 2,441 cases have 3 controls, and 8,324 cases have 4 controls

^7^ 2,640 cases have 1 control, 3,789 cases have 2 controls, 2,544 cases have 3 controls, and 7,481 cases have 4 controls

^8^ *p*-values for the stratified analyses are shown in the parentheses

## Discussion

In this study, sow mortality was observed to increase generally during the 6-year period from 2019 to 2024 in Spain. This trend is consistent with a nationwide Spanish study spanning 109–123 pig production companies, which similarly reported a steady rise in sow mortality from 2015 to 2024, from approximately 8–9% up to around 14–15% [4]. A comparable upward trend has also been reported internationally; for example, mortality rates in the United States rose from 12.31% in 2019 to 14.67% in 2023 [47].

Regarding the timing of sow deaths, mortality incidence was higher post-farrowing than during gestation. Peak incidence rates observed between 105 and 118 days of gestation (near farrowing), and in the first weeks after farrowing, aligning with findings from previous studies [12, 40]. Notably, a sharp increase in mortality incidence was observed after 21 days post-farrowing (Table 6), a pattern also reported in studies of lameness and prolapse incidence [27, 48]. Because most sows are weaned and rebred by the fourth week post-farrowing, the marked decline in sow-years at risk after week 4 (Table 6) suggests that the sows remaining at risk beyond this point represented a selected subgroup, likely including animals with reproductive problems, illness, or no further breeding intent, rather than a random subset of the population. A second rise in mortality incidence at 7 weeks post-farrowing followed a similar pattern: the predominant causes of death during this period were accidents, moribund conditions, and illness rather than prolapse (Fig. 6), indicating that this later peak was likewise more plausibly driven by reproductive failure, health problems, or exclusion from further breeding, rather than by the post-weaning period itself.

In other studies, the peripartum period was reported to account for more than 50% of all sow mortality cases [12, 19, 49], and the possible reasons are heart failures, genitourinary lesions, and prolapses based on post-mortem diagnosis [12]. Necropsy-based investigations have further revealed that mortality in sows often involves diverse underlying conditions. For example, one study reported positional changes in internal organs (32% of 100 deaths), arthritis (19%), and urogenital disorders (7%) as the most frequent findings in dead sows [50]. Several stressors, such as heat stress, aggressive interactions, and increased body weight, have been identified as contributing factors to peripartum mortality, particularly due to heart failure [15, 49, 51, 52]. In addition to cardiovascular issues, urinary tract infections are a leading global cause of culling and sudden death in sows [53]. Heavier pigs and those with lameness issues have a higher likelihood of ascending infections because their lower body parts are more likely to come into contact with surfaces contaminated by feces [54, 55], while sows in late gestation due to their increased weight have more likelihood to urinary tract infections. Floor types that have better drainage effects should be considered. Both gestation and farrowing are significant risk factors for prolapse, as the reproductive process weakens connective tissues, making prolapse a major concern. Studies have identified parity, litter size, and piglet birth weight as contributing factors, with pelvic organ misalignment resulting from structural weakening [56]. In humans, pelvic organ prolapse has been linked to low bone mineral density [57], and postmenopausal women are at higher risk due to estrogen deficiency. Similarly, among late-gestation sows, those with lower body condition scores have shown a higher incidence of prolapse compared to those in normal or overweight conditions [58]. Given these findings, optimizing prenatal nutrition, particularly calcium supplementation, may help reduce the risk of prolapse.

In our study, mortality incidence declined progressively from farrowing through the second week postpartum, before rising again from around 21 days post-farrowing (182.22 per 1,000 sow-years), coinciding with the weaning period (21–27 days post-farrowing), during which sudden death was the most frequently recorded cause of death. To gain deeper insight, we referred to a previous study that reported a 5.35% mortality rate (6 out of 112 sows) during the weaning-to-estrus interval. In that study, confirmed causes of death included heart failure (2 cases), liver lobe torsion (2 cases), and vaginal or rectal prolapse (2 cases) [32]. Notably, two-thirds of these cases could potentially explain some of the sudden deaths observed in our study. However, due to the small sample size of that study, it does not fully capture the range of potential causes during this period, nor does it clarify the true composition of sudden deaths in our dataset. Several factors may contribute to increased mortality at weaning, including the stress of separation from piglets, hormonal fluctuations associated with the return to estrus, and stress induced by relocation. These factors could exacerbate underlying health conditions, ultimately leading to sudden death.

For the herd-level risk factors, although internal gilt replacement is commonly associated with enhanced biosecurity, our findings indicate that farms employing this strategy had higher sow mortality incidence rates after farrowing and throughout the reproductive cycle compared with farms that purchase external replacement gilts. Importantly, this association was not confounded by herd size or productivity (PWSY), as neither factor showed a significant relationship with mortality in our data. This suggests that the elevated risk may stem from other herd-level dynamics. One plausible explanation is that internal replacement systems place additional demands on gilt development and selection, and the present study did not directly assess factors such as gilt body condition, or genetic diversity within closed herds; these remain candidate mechanisms that could be explored in future studies incorporating replacement-specific management data.

Our results indicate that sows beyond parity 6 face the highest overall mortality risk, likely due to age-related health deterioration and increased susceptibility to disease. High parity sows had a higher mortality incidence rate after service and a rather lower incidence rate after farrowing than other parities. Meanwhile, parity 1 sows also experienced elevated mortality, primarily due to a higher post-farrowing death rate than in any other parity. This may stem from the physiological immaturity of young sows, as their bodies are still developing and may not yet be fully prepared for pregnancy and farrowing. Mortality incidence declines with parity, reaching its lowest point at parity 4, before rising again in later parities. In contrast, pre-farrowing mortality is lowest in parity 1 sows but increases steadily with advancing parity.

Mortality risk varies across studies depending on the parity cycle phase but is generally highest in low-parity sows or those at parity 6 and beyond [1, 5, 40, 49, 59–61]. One possible explanation for this discrepancy is differences in culling strategies across studies. In our study, farms may have lower culling efficiency for older sows, meaning that more high-parity sows remain in the herd until they die, rather than being removed pre-emptively. In contrast, studies with more aggressive culling policies may observe fewer deaths in older sows simply because they are removed before reaching a critical state of health.

Service history appears to be associated with differential mortality risk across reproductive stages. Sows that underwent repeat services exhibited a lower mortality incidence during the gestation period. One possible explanation is that the additional time between services may allow these sows more opportunities to recover physically before conceiving again. However, previous studies have associated a longer weaning-to-service interval with increased culling rates and reduced farrowing proportions [62, 63]. This may be because in those studies, sows with poorer reproductive health either failed to conceive at the first service or were deliberately delayed by farm managers, reflecting underlying health concerns that predisposed them to culling or reproductive inefficiency. Since our study focuses exclusively on mortality (not culling), the lower mortality observed in repeat-service sows may partly reflect a survivorship bias, that the sows with evident reproductive issues might have already been removed from the herd before death. Supporting this interpretation, our post-farrowing mortality analysis revealed that sows with repeat services had a higher incidence of death after farrowing, suggesting that reproductive dysfunctions reflected by repeat services may manifest more clearly as health risks in the postpartum period.

Several studies have established that high mortality rates in both winter and summer are an industry-wide concern [49, 59, 60, 64–67]. In the results, sows bred in spring and those farrowing in summer had the highest mortality incidence rates. Given that pre-farrowing deaths were concentrated in the final one to two weeks of gestation, the mortality peak for spring-bred sows still occurred in summer. This temporal pattern is consistent with a role for summer heat stress in driving sow mortality, although the present study did not directly measure in-barn temperature, and farm-level differences in ventilation and cooling system design, which can substantially decouple indoor conditions from ambient outdoor temperature, were not accounted for. As such, the extent to which external climatic conditions translated into actual heat exposure for sows in each farm remains unknown, and this explanation should be regarded as plausible rather than established.

Notably, since 2022, summer heat waves have been increasingly reported across Europe [68, 69]. Spain, located in the southern Europe, suffered the most from the global warming among European countries because of its drought in summer [70, 71]. According to the State Meteorological Agency (AEMET), 2023, 2022, and 2024 were the first, second and third-hottest years [72–75].Most of the farms included in the studies were from the north east of Spain, including Catalonia, Aragon and Navarre. This part of Spain experienced the hottest summer in 700 years in 2022 [76]. These heat waves have caused numerous human fatalities [77] and, unsurprisingly, also significantly impacted livestock production [78]. While these external climatic records coincide with the marked mortality peak observed after 2022 in our summer data (Fig. 3), confirming a direct causal link would require farm-level indoor temperature data, ideally obtained from climate control systems, to disentangle the contribution of ambient heat from other co-occurring seasonal factors.

Although no pathogen surveillance was conducted, infectious disease may have contributed to the observed mortality patterns. Sows that died had more stillbirths than their matched controls, a finding compatible with reproductive infectious diseases, including PRRSV, which is a well-recognized cause of stillbirths and increased sow mortality. This interpretation is further supported by the change in PRRSV epidemiology in Spain since the introduction of the Rosalia strain in 2020. This new strain causes severe outbreaks with high impacts on production and farm economics, including substantial sow losses, abortion rates of up to 27% sustained over 17 consecutive weeks, and piglet nursery mortality peaking between 28% and 50% [79]. The wider impact, including on sow mortality, of this strain on the Spanish swine industry has been reported by Font et al. [4]. We further speculate that the recurrent winter mortality peaks observed after 2022 (Fig. 3) could be related to PRRSV transmission dynamics, given the virus’s greater environmental viability under cold conditions [80–82]. Nevertheless, the absence of diagnostic confirmation precludes attributing the observed patterns specifically to PRRSV.

Beyond infectious agents, several physiological and environmental stressors also contribute to stillbirths and sow mortality. Risk factors associated with stillbirths, such as higher parity, larger litter size, and prolonged farrowing duration, contribute to increased reproductive stress, which in turn elevates the risk of sow mortality [83–87]. Also, heat stress not only increases the likelihood of stillbirths but is also recognized as a major contributor to sow mortality [85]. The impact of body condition score is another critical factor linked to both stillbirth rate [83, 85] and mortality risk [12, 88, 89]. In addition, management-related factors such as restrictive farrowing crates, poor locomotion, feed refusal, and inappropriate timing of crate entry increase stillbirth risk [90].

Therefore, the elevated number of stillbirths observed in deceased sows is unlikely to be an isolated phenomenon. Rather, it may signal a broader syndrome of both stress-related and infectious factors, underlining the need for integrated monitoring of both sow health and reproductive performance to reduce perinatal losses and improve sow longevity.

In both reproductive and lifetime performance, dead sows tended to have a younger age at first service, while previous studies have suggested that an earlier first farrowing is linked to increased longevity [9, 18, 29, 91, 92]. Survival analysis of reproductive failures showed that a later age at first farrowing significantly heightened the risk of culling [93]. While this finding seems to contradict our results, it is important to note that our study specifically examined sow mortality, without accounting for culling events or the overall length of sows’ productive life on the farm.

The average parity of dead sows is 3.42, significantly lower than the average parity at removal for non-mortality sows (shown in Table 8). This finding aligns with previous research, which reported an average parity at death ranging from 3.4 to 4.3 [13, 94]. Notably, sows must remain in the breeding herd for at least 3 to 4 parities to offset their initial production costs [95, 96]. Therefore, reducing sow mortality and extending sow longevity are critical for maintaining economic sustainability and herd productivity.

A comparison of the average number of piglets born per parity over a sow’s lifetime between deceased sows and their matched controls revealed that sows that died had larger litters and more live-born piglets on average, and they also exhibited higher numbers of stillbirths and mummified fetuses. The increased litter size and live-born count suggest that deceased sows had superior reproductive performance, which may be linked to their younger age at first service, a factor associated with improved reproductive outcomes in multiple studies [18, 29, 97, 98].

However, the elevated stillbirth and mummified fetus rates contradict this interpretation. Instead, these findings likely indicate that an increase in stillbirths and mummified fetuses serves as a warning sign of impending mortality. It might be speculated that infectious pressures, such as PRRSV outbreaks, could underline part of this association, since PRRSV is known to increase both sow mortality and the incidence of stillbirths and mummified fetuses.

Our study has several limitations. Although the weaning period showed a mortality peak, the lack of weaning date records prevented us from using weaning as a reference point in the analysis. In addition, causes of death relied on farm-recorded classifications without post-mortem confirmation, which may have resulted in misclassification and reduced the reliability of cause-specific analyses. Furthermore, the absence of comprehensive breed and farm management data limited our ability to evaluate additional risk factors. Despite these limitations, this study provides valuable insights for industry professionals.

## Conclusions

This study identified the periparturient period as the period of highest mortality risk for sows. Mortality patterns were associated with both herd-level management factors and individual sow characteristics. Internal gilt replacement was associated with higher mortality compared to external replacement, and a U-shaped relationship was observed between parity and mortality risk, with the highest risk concentrated at both low and high parities. Mortality was also higher among sows bred in spring and farrowing in summer, while repeat breeding prior to farrowing was associated with lower mortality.

Findings from the matched case-control analyses indicated that sows that died had shown early-life reproductive disadvantages despite higher fetal outputs later in life, suggesting a disconnect between measures of productive output and long-term sow sustainability. Together, these findings support incorporating reproductive history and herd-level replacement strategy into future risk assessment and culling decision frameworks. Further research is needed to clarify the underlying physiological and management mechanisms driving these associations.

## Data Availability

The datasets used and/or analyzed during the current study belong to the private company. They are not publicly available.

## Abbreviations

PWSY: Pigs weaned per sow per year
CI: Confidence interval
SD: Standard deviation
IQR: Interquartile range
PRRSV: Porcine reproductive and respiratory syndrome virus
